# Spatially Organized DNA-Templated Silver Nanoclusters as Potent Antimicrobial Agents for ESKAPE Infections

**DOI:** 10.1021/acsami.5c25898

**Published:** 2026-03-16

**Authors:** Elizabeth Skelly, Krishna Majithia, Laura P. Rebolledo, Camila Fonseca Rizek, Silvia Figueiredo Costa, Alora R. Dunnavant, Cheyenne Vasquez, Alexander J. Lushnikov, Alexey V. Krasnoslobodtsev, Taejin Kim, Morgan R. Chandler, Renata de Freitas Saito, Roger Chammas, M. Brittany Johnson, Kirill A. Afonin

**Affiliations:** † Chemistry and Nanoscale Science Program, Department of Chemistry, 14727 University of North Carolina at Charlotte, Charlotte, North Carolina 28223, United States; ‡ Department of Biology, University of North Carolina at Charlotte, Charlotte, North Carolina 28223, United States; § Laboratório de Investigação Médica 49, 28133 Departamento de Infectologia e Medicina Tropical da Faculdade de Medicina da Universidade de São Paulo, Av. Dr. Eneas de Carvalho Aguiar, 470, São Paulo 05403-000, Brazil; ∥ Department of Physics, 14720 University of Nebraska Omaha, Omaha, Nebraska 68182, United States; ⊥ Department of Physical Sciences, West Virginia University Institute of Technology, Beckley, West Virginia 25801, United States; # Centro de Investigação Translacional em Oncologia (LIM24), Departamento de Radiologia e Oncologia, Faculdade de Medicina da Universidade de São Paulo and Instituto do Câncer do Estado de São Paulo, São Paulo, São Paulo 01246-000, Brazil; ¶ Centres for Antimicrobial Optimisation Network (CAMO-Net) Brazil, Faculty of Medicine, University of São Paulo, São Paulo 05403-010, Brazil; ∇ MIMETAS US, Inc, Gaithersburg, Maryland 20878, United States

**Keywords:** silver nanoclusters, ESKAPE pathogens, bone infection, staphylococcus aureus, resistant bacteria

## Abstract

Antibiotic-resistant bacteria cause more than one million deaths annually worldwide. The rapid evolution and horizontal gene transfer among pathogens frequently render newly developed antibiotics ineffective shortly after their introduction, underscoring the urgent need for alternative therapeutic strategies. Nanoscale silver is well known for its innate antimicrobial activity but typically requires high concentrations for efficacy that causes toxicities and limits broader clinical applications. To overcome these limitations, we introduce programmable, self-assembling DNA scaffolds that template, stabilize, and spatially organize multiple copies of monodisperse silver nanoclusters (DNA-AgNCs). These nanoscale assemblies enhance the antimicrobial potency of formulations while exhibiting intrinsic fluorescence, providing a dual functionality for therapeutic and fluorescence probing applications. Comprehensive characterization revealed DNA-AgNCs with superior stability and potent activity against clinically relevant antibiotic-resistant ESKAPE pathogens. Also, DNA-AgNCs significantly reduced the intracellular bacterial burden in primary murine bone cells infected with *Staphylococcus aureus*. Mechanistic studies indicate that bacterial killing by DNA-AgNCs is mediated by reactive oxygen species, particularly singlet oxygen, in conjunction with the disruption of the bacterial membrane. Furthermore, DNA-AgNCs retained strong antibacterial activity after 4 weeks of storage at ambient temperatures, with minimal loss of efficacy. Collectively, these findings establish spatially organized DNA-AgNCs as a promising, modular platform for next-generation antibacterials with integrated real-time fluorescence probing capabilities.

## Graphical abstract

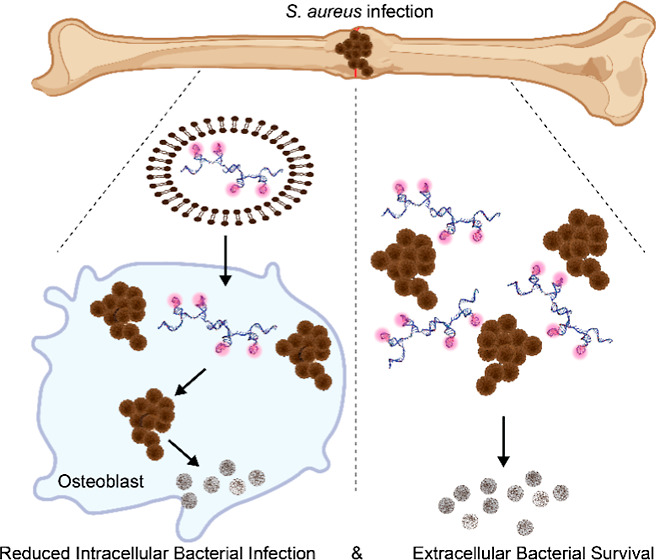

## Introduction

The rise of multidrug-resistant bacterial species has made antibiotic resistance a pressing global health and economic crisis that must be addressed through the development of novel antibacterial therapies capable of halting the pathogenesis of systemic bacterial infections.^1,2^ Among the most clinically significant organisms are ESKAPE pathogens, including *Enterococcus faecium* (*E. faecium*), *Staphylococcus aureus* (*S. aureus*), *Klebsiella pneumoniae* (*K. pneumoniae*), *Acinetobacter baumannii* (*A. baumannii*), *Pseudomonas aeruginosa* (*P. aeruginosa*), and *Enterobacter* spp., which are known for their ability to resist killing by currently available antibiotics. *S. aureus* is especially concerning due to its ability to cause a wide range of infections, from superficial skin and soft tissue infections to life-threatening diseases such as sepsis, endocarditis, and osteomyelitis.^3,4^

Nearly 80% of osteomyelitis cases result from a severe manifestation of *S. aureus* infection and is associated with prolonged illness, disability, and in some cases, mortality.^5,6^ Treatment is challenging due to the multiple strategies that *S. aureus* employs to evade immune defenses and antimicrobial therapies.^4,7^ Externally, the bacteria form biofilms on necrotic bone tissue, creating a protective matrix and shielding itself from antibiotics and immune clearance.^8,9^ Internally, *S. aureus* invades resident bone cells, including osteoblasts, osteocytes, and osteoclasts, as demonstrated by both in vitro and in vivo studies.^10–17^ Once internalized, bacteria can persist as an intracellular reservoir, evading killing by most host immune cells and antibiotics.^7,13,18–20^ These features contribute to recurrent and chronic osteomyelitis and highlight the urgent need for alternative therapeutic strategies targeting *S. aureus*.

One possible therapeutic strategy involves combining the therapeutic properties of silver, a historically renowned antibacterial agent, with nanotechnology, thus engineering novel nanoscale silver formulations for advancing modern disease prevention.^21,22^ These silver nanoparticles possess antimicrobial modalities that bacteria have difficulty evading and are one of the most widely accepted antibacterial nanoagents both in a clinical setting and across a multitude of consumer products (medical implants, wound dressings, textiles, cosmetics, etc.).^22–26^ Although novel and clinically accepted, the applications of silver nanoparticles are limited by poorly understood mechanisms and toxicity to mammalian cells.^27–30^ Due to these concerns, the use of silver nanoparticles is currently restricted to surface-level or localized therapeutics only. They have not expanded as clinically acceptable treatment methods for bacterial infections, such as osteomyelitis.

DNA-templated and -stabilized silver nanoclusters (DNA-AgNCs) are a new promising alternative therapeutic that could expand the applicability of nanoscale silver.^31–34^ Silver ions have a high affinity for cytosine bases.^35^ Cytosine-rich ssDNA sequences thus define tunable binding sites that template and stabilize AgNC formation upon reduction.^31,36–38^ These atomically precise hybrid biomolecule-metal nanostructures typically contain 5–30 silver atoms, giving them unique and tunable optical properties with advantageous antibacterial activity.^39–45^ The unique fluorescence of DNA-AgNCs arises from their ultrasmall size as well as the templating oligonucleotide’s structure and complementary base interactions associated with the primary structure. The specific fluorescence color and excitation–emission patterns depend on the stabilizing ssDNA oligomer sequence, size, shape, and the number of neutral silver atoms (Ag^0^) per cluster.^37,38,45–51^ The antibacterial properties of this therapeutic have been shown to be effective against both Gram-negative and Gram-positive bacteria.^28,31–34,52^ However, the application of DNA-AgNCs in treating bacterial infections in mammalian model systems remains unexplored, thus presenting a promising path for future research and discovery.

Our previously published study demonstrated that DNA-hairpins with varying cytosine counts in the hairpin loop displayed the antibacterial activity against *Escherichia coli* (*E. coli*).^29,31^ While DNA-AgNCs have been evaluated against various Gram-positive and Gram-negative bacteria, these studies have focused on planktonic bacteria and DNA-AgNCs effectiveness against specific strains in infected mammalian cells.^32,44,53,54^

In the current study, we screened a panel of 11 distinct DNA-AgNCs, each templated on varying numbers of single-stranded cytosines within hairpin (HP) structures ranging from C5 to C15, and identified the C13 HP as the most stable, optimal for fluorescence probing, and effective antimicrobial agent against clinically relevant, antibiotic-resistant bacterial strains. We demonstrated that combining multiple C13 HPs into single-stranded DNA scaffolds, as well as into multistranded fibrous DNA supra-assemblies, significantly enhances the antimicrobial activity of formulations. Furthermore, we identified multiple mechanisms by which DNA-AgNCs mediate bacterial killing. Both C13 DNA-AgNCs and C13-based fibrous DNA-AgNCs were successfully delivered into mammalian cells, where they restricted the intracellular bacterial burden. Collectively, these findings demonstrate that increasing the higher number of DNA-AgNCs within a single structure enhances antibacterial efficacy, while their therapeutic concentrations can be optimized to minimize toxicity to mammalian cells.

## Results and Discussion

### DNA-AgNCs Display Significant Antimicrobial Activity Against Planktonic *S. aureus*

DNA HPs with cytosine-rich loops have been demonstrated to template DNA-AgNCs, which have gained interest due to their fluorescent and antibacterial properties.^30,36,55–57^ However, effective antibacterial activity typically requires high concentrations, which may also impact mammalian cells. To address this limitation, the goal of this work was to engineer DNA-AgNCs with increased antibacterial activity and reduced toxicity.

DNA HPs with single-stranded C5–C15 in the loop were used as scaffolds to synthesize 11 distinct DNA-AgNCs (Figure 1a). Each sample exhibited unique fluorescence under UV excitation (340 nm), following a reverse rainbow order: the smallest HP loops (C5 and C6) produced faint blue fluorescence, while the largest HP loops (C13 to C15) produced red fluorescence (Figure 1b). The number of silver atoms per HP structure was quantified by using energy-dispersive X-ray spectroscopy (EDS; Figure 1c). C13 DNA-AgNCs exhibited the highest number of silver atoms per hairpin loop compared with the C5–C15 variants. Notably, the silver atom count increased progressively from C5 to C13, followed by a decrease for C14 DNA-AgNCs. In addition, the number of silver atoms in C15 DNA-AgNCs was closer to that observed for C12 than for C13. To evaluate their optical properties, excitation–emission spectra were recorded 24 h after synthesis, revealing that each sample possessed unique excitation and emission maxima, as shown in (Figure 1d). Across all samples, fluorescence was the most intense under yellow-to-red visible light excitation.

**Figure 1:**
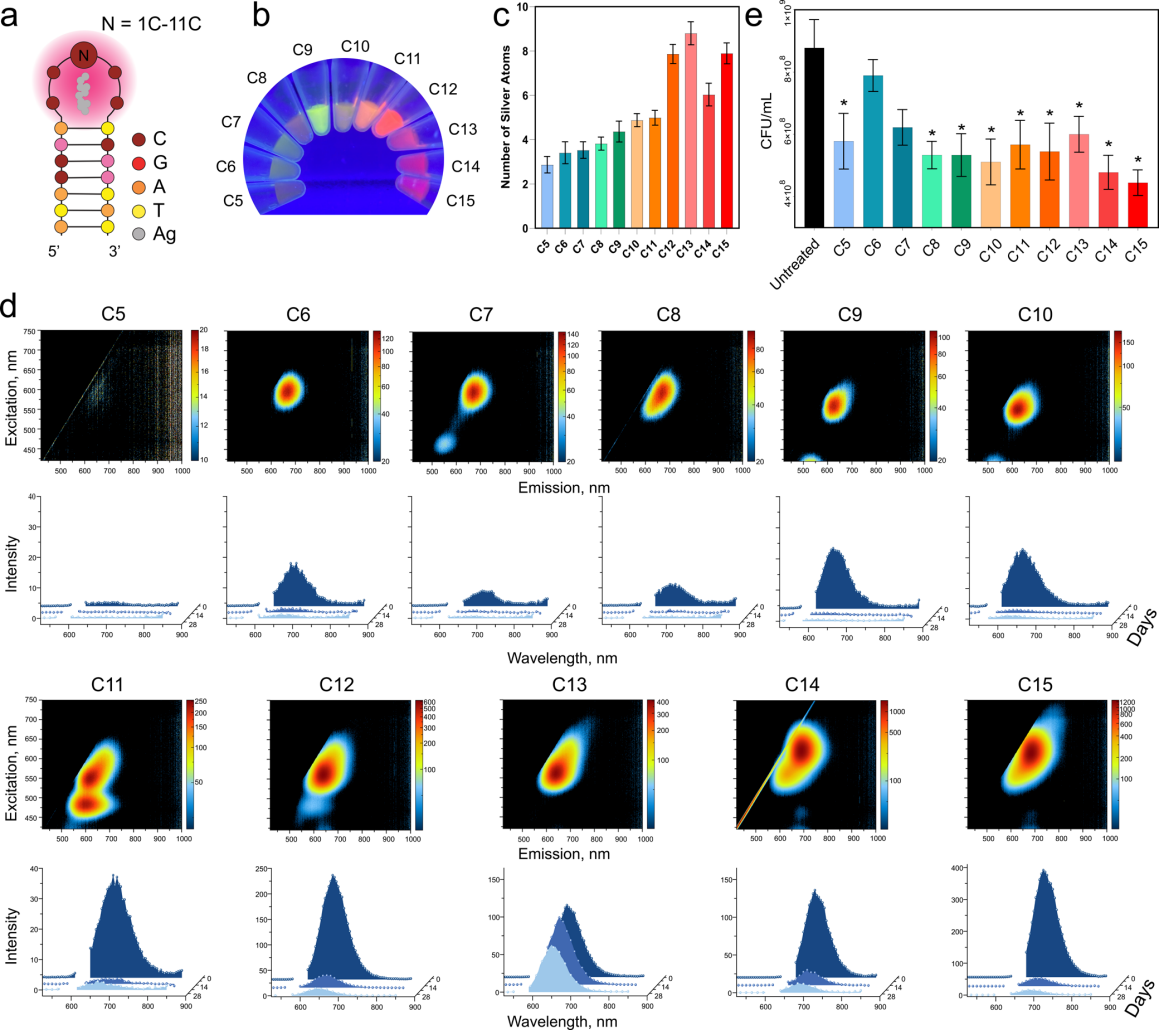
Evaluation of physical and biological property changes in DNA hairpins (HPs) with 5 to 15 cytosines in the loop (C5–C15). (a) Schematic representation of DNA HPs with a consistent stem region and varying numbers of cytosines. (b) Fluorescence intensity differences among DNA-AgNCs at 25 μM DNA. (c) Number of silver atoms per AgNC determined by energy dispersive X-ray spectroscopy (EDS). Error bars show mean ± SEM, *n* = 4. (d) Initial excitation–emission matrices and changes in fluorescence over 4 weeks for DNA-AgNCs at 10 μM DNA. (e) Colony-forming units (CFUs) of *S. aureus* treated with DNA-AgNCs at 4 μM DNA, assessed 6 h post-treatment. Error bars represent mean ± SEM, *n* = 3, *P < 0.05.

To assess the stability of DNA-AgNCs, excitation–emission spectra were recorded 2 and 4 weeks after synthesis, while the samples were stored in the dark at 4 °C. Over time, most emission peaks decreased in intensity, indicating gradual aging of the nanoclusters, although certain designs exhibited greater stability than others. Notably, C13 DNA-AgNCs maintained a consistent emission signal throughout the storage period (Figure 1d), indicating the most stable conformation for use in the following studies. We observed an increase in fluorescence intensity with an increase in the number of silver atoms.

The antibacterial activity of all DNA-AgNCs was compared against the clinically relevant *S. aureus* strain, UAMS-1. Among these, C5 and C8–C15 DNA-AgNC structures exhibited significant antibacterial effects compared with untreated controls (Figure 1e), with no major differences observed between the structures. Interestingly, the degree of antibacterial activity did not show any correlation with a wide range of emissions. In contrast, C6 and C7 exhibited negligible antibacterial activity despite displaying higher fluorescence intensities in the excitation–emission spectra. In general, samples containing fewer silver atoms per hairpin exhibited reduced antibacterial activity.

### Increasing the Number of C13 Hairpin Loops on Nanostructures Enhances the Antimicrobial Activity Against Planktonic *S. aureus*

To enhance antibacterial activity and reduce the concentration required to kill *S. aureus*, we increased the number of DNA-AgNCs within a single nanostructure, thereby increasing the local concentration of silver. Based on the unique excitation emission spectra for each of the C5–15 hairpin structures and their relative stabilities, we selected the C13 hairpin to proceed with. The C13 hairpin has an emission in the red wavelength range which is ideal for fluorescence probing and maintains higher stability over time when compared to all other HPs.

We next tested single-stranded multihairpin structures containing either two or three C13 loops, hereafter referred to as 2HP and 3HP, respectively. To assess the effect of structural flexibility, additional 3HP variants were designed with one, two, and three thymine spacers inserted between the adjacent HPs. Computational modeling supported these designs (Figure 2a,b), while a single C13 hairpin was used as a reference control. Both 2HP and 3HP structures exhibited fluorescence comparable to that of a single C13 hairpin (Figure 2c,d). Notably, the 3HP structure showed the highest stability among the constructs, with the smallest decrease in fluorescence over time.

**Figure 2:**
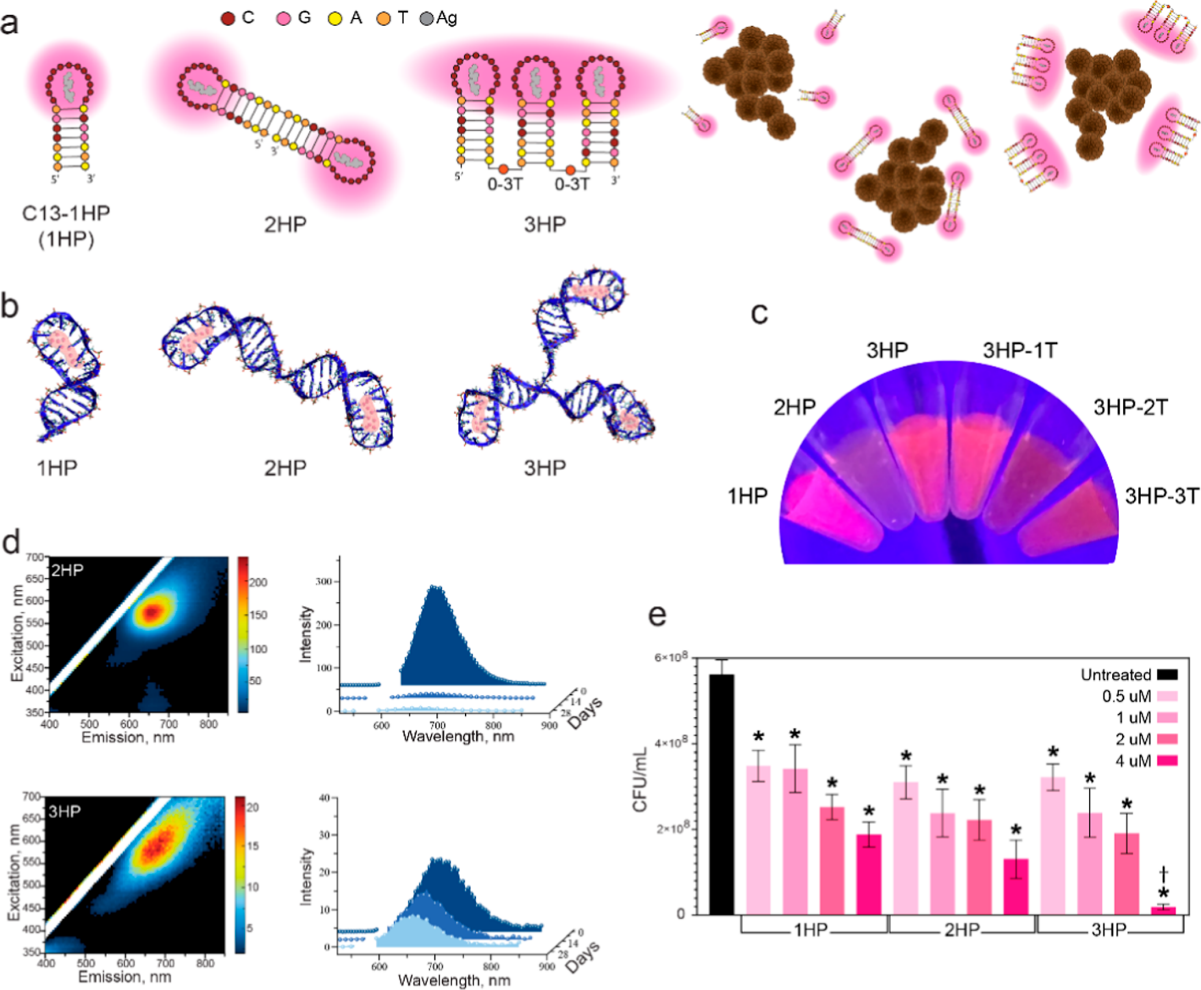
Evaluation of physical and biological property changes in multihairpin DNA-AgNCs. (a) Schematic of multihairpin DNA-AgNCs enabling more silver to surround bacteria at equimolar DNA concentration. (b) Computational models of multihairpin DNA-AgNCs. (c) Visual appearance of multihairpin DNA-AgNCs under UV excitation. (d) Initial excitation–emission matrices and fluorescence intensity changes over time of multihairpin DNA-AgNCs at 10 μM DNA. (e) Colony-forming units (CFUs) of *S. aureus* treated with multihairpin DNA-AgNCs at varying concentrations (0.5, 1, 2, 4 μM) of DNA, assessed 6 h post-treatment. Error bars represent mean ± SEM, *n* = 9, *P < 0.05. Dagger denotes a significant drop in CFU for the 3HP sample, as compared to the 1HP and 2HP samples at 4 μM.

Next, we assessed if increasing the local concentration of silver influences the antibacterial activity against planktonic *S. aureus* across a concentration range of 0.5 to 4 μM DNA. We observed a clear dose-dependent reduction in viable bacteria with maximal bacterial death at 4 μM DNA. Notably, samples containing a greater number of hairpin structures demonstrated a further reduction in bacterial viability at 4 μM DNA, indicating that higher localized silver loading enhances the antibacterial activity of these nanostructures (Figures 2e, and S1).

Also, samples were compared at equivalent silver concentrations using DNA-AgNCs and silver nitrate (AgNO_3_). Although AgNO_3_ was effective, its antibacterial activity was not significantly different from that of DNA-AgNCs (Figure S1), suggesting that free silver ions contribute to the observed antibacterial effects. However, AgNO_3_ alone is not suitable for therapeutic use due to its toxicity toward mammalian cells.^58^ In contrast, 10 and 50 nm silver nanoparticles showed no detectable bactericidal activity at the highest concentrations tested. Thus, incorporation of silver into DNA-based architectures not only maintains high antibacterial efficacy but also enhances biocompatibility, and increasing the number of hairpins per DNA scaffold raises the number of active pharmaceutical ingredients (API) per excipient.^59^

We further determined the minimal inhibitory concentration (MIC) and the minimal bactericidal concentration (MBC) of the 3HP DNA-AgNCs against clinically relevant antibiotic-resistant Gram-negative (*A. baumannii*, *P. aeruginosa*, and *K. pneumoniae*) and Gram-positive (*S. aureus* and *E. faecalis*) bacterial species (Figures 3, S2 and S3). All of the tested bacterial species are recognized as public health threats. According to the Center for Disease Control and Prevention (CDC) Report on Antibiotic Resistance Threats in the United States,^60^ Carbapenem-resistant *Acinetobacter* is an urgent threat, while Vancomycin-resistant *E. faecalis* (VRE), Multidrug-resistant *P. aeruginosa* and Methicillin-resistant *S. aureus* (MRSA) are considered serious threats. Similarly, Carbapenem-resistant *K. pneumoniae* is included in the World Health Organization (WHO) list of emerging bacteria that pose the greatest threat to human health.^61^

**Figure 3:**
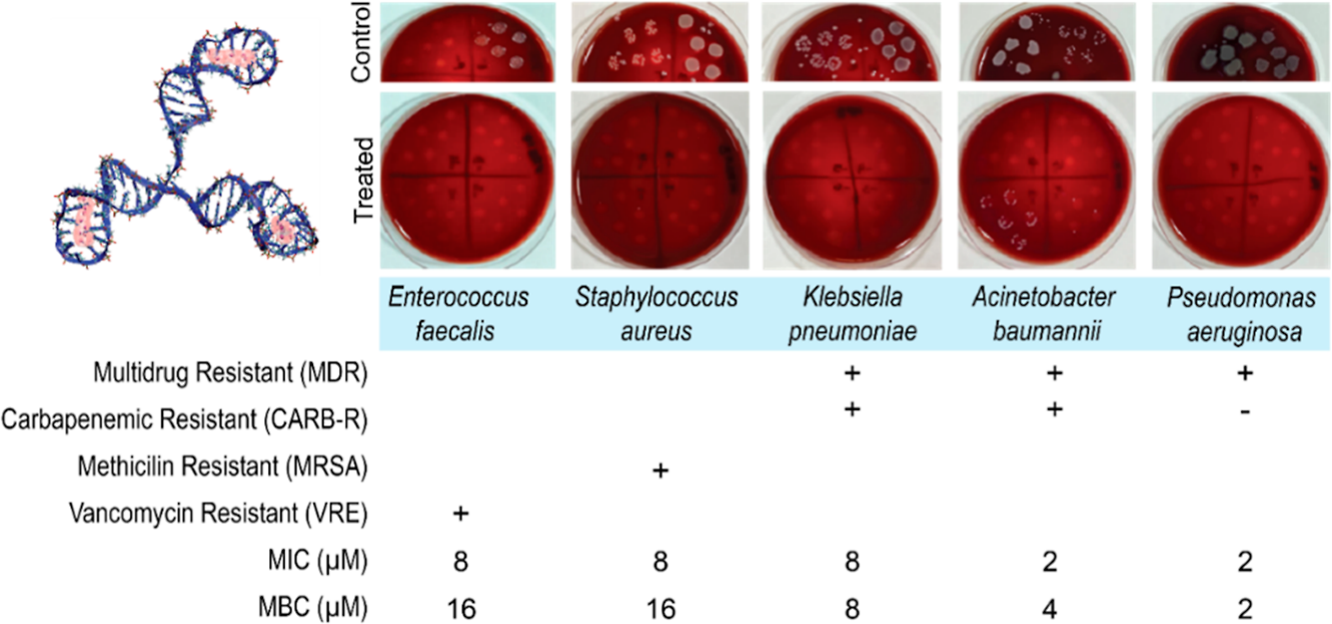
Evaluating C13–3HP DNA-AgNCs (3D model is shown in the top left) against drug-resistant Gram-negative (*A. baumannii*, *P. aeruginosa*, *K. pneumoniae*) and Gram-positive (*S. aureus* and *E. faecalis*) bacteria, with representative plates for each control and treated bacteria used for counting viable CFU. The specific drug resistance is noted by (+), with minimum inhibitory concentrations (MIC) and minimum bactericidal concentrations (MBC) listed. Entire pictures of the control plates are shown in Figure S3.

The 3HP DNA-AgNCs displayed broad antimicrobial activities against all antibiotic-resistant bacteria. Resistant clinical strains showed MICs of 2–4 μM for Gram-negative and 8–16 μM for Gram-positive isolates, except for *K. pneumoniae*, which exhibited a higher MIC of 8 μM compared to the Gram-negative ATCC strains. In contrast, susceptible ATCC strains displayed MICs of 1–4 μM for Gram-negative bacteria and 4–8 μM for Gram-positive bacteria (Table S2). Overall, Gram-positive bacteria showed higher MICs than Gram-negative. For MBCs, Gram-negatives matched their MICs across ATCC and clinical strains, whereas Gram-positives consistently required one dilution higher (8 μM for ATCC, 16 μM for resistant strains). This is consistent with previous studies demonstrating that silver nanoparticles were more effective against Gram-negative bacterial species.^62^ The MBC/MIC ratio, with values below 4 indicating bactericidal activity,^63^ suggests that DNA-AgNCs confer bactericidal effects. The observed differences in sensitivity of Gram-negative and Gram-positive bacteria may be in part due to the differences in bacterial structures. In contrast to Gram-negative bacteria, which have a thin peptidoglycan cell wall and an additional lipid outer membrane, Gram-positive bacteria have a thick peptidoglycan cell wall, which may provide a barrier hindering the permeation of Ag^+^ ions through the cytoplasmic membrane.^64,65^ The consistent overlap of MIC and MBC values in Gram-negative bacteria suggests rapid bactericidal activity, while the one-dilution gap in Gram-positive bacteria implies a slower or less efficient killing process, possibly due to cell wall barriers limiting the effective intracellular accumulation of the nanoclusters. Using silver concentration as the API, we calculated the MIC for 3HP DNA-AgNCs and compared to standard antimicrobial agents (Table S3). DNA-AgNCs exhibited up to 78-fold greater antibacterial potency compared with standard antibiotics (*e*.*g*., amikacin).

### Fibrous DNA-AgNCs Display Antimicrobial Activity Against Planktonic *S. aureus*

After the successful synthesis and characterization of multiple hairpin DNA-AgNCs, we engineered fibrous DNA-AgNCs. Fibrous DNA-AgNCs were assembled from two monomers per repeat, each containing one or two hairpins that mimic the C13 single-hairpin structure (Figure 4a,d). Additionally, the fibrous DNA-AgNCs were designed to have varying degrees of flexibility, with thymine linkers inserted between the adjacent HPs monomers, and the resulting structures were computationally modeled. Like the single and multihairpin C13 constructs, the fibrous DNA-AgNCs exhibited red fluorescence (Figure 4b) maintaining their potential use for fluorescence probing applications. Notably, our results suggest an inverse relationship between the structure flexibility and fluorescent stability. Stability testing showed that fibrous DNA-AgNCs with a single hairpin on each monomer had the most consistent excitation–emission spectra over time. The fibrous DNA-AgNCs with two hairpins on each monomer with the least amount of flexibility were found to be the most stable overtime as indicated by the excitation–emission spectra (Figure 4c). AFM imaging confirmed the formation of fibrous DNA-AgNCs (Figure 4e), which is consistent with the predicted designs. The AFM images show continuous, elongated features indicative of a one-dimensional self-assembly of DNA scaffolds that spatially organize the incorporated AgNCs along the fiber axis, which we refer to as a “fibrous” morphology. Quantitative analysis further shows that the observed fiber lengths extend well beyond the expected monomeric size of a few nanometers. The longest observed fibers range between 43 nm (for 2HP-2T-F) and 75 nm (2HP-0T-F), with intermediate lengths of 57 nm (2HP-1T-F and 2HP-3T-F) and 66 nm (1HP-F).

**Figure 4:**
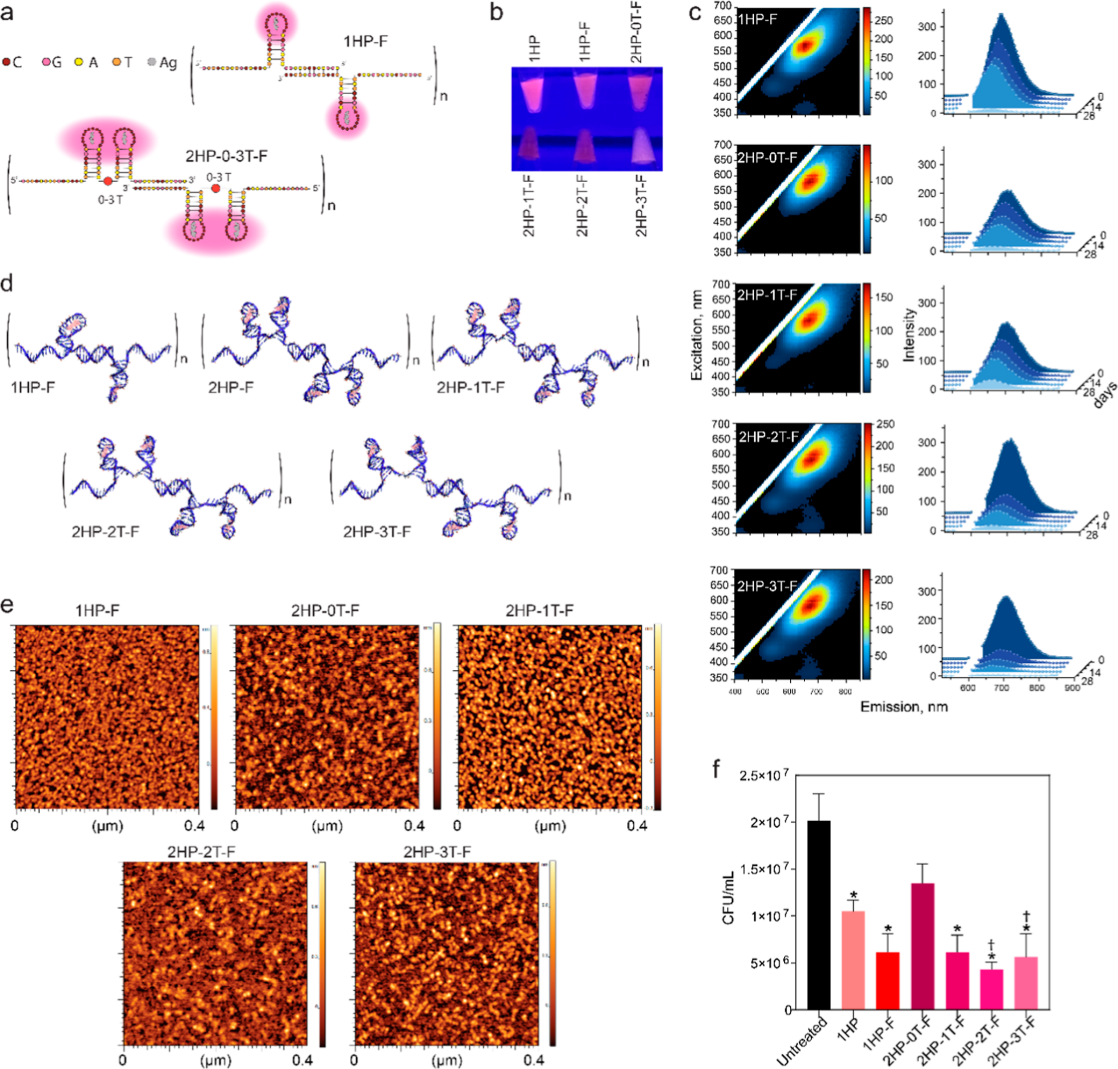
Evaluating the change in physical properties and biological activity of DNA-AgNCs when hairpin structures are introduced on fiber structures. (a) The depiction of fibrous DNA-AgNCs that either have a single hairpin on each monomer (e.g., 1HP-F) or two hairpins on each monomer with 0–3 thymines between the two fibers (e.g., 2HP-0T-F). (b) The colors of fibrous DNA-AgNCs when excited with UV light, as compared to 1HP DNA-AgNC. (c) Excitation–emission matrices for fibrous DNA-AgNCs at 10 μM DNA. (d) Computational models of fibrous DNA-AgNCs. (e) AFM images of the fibrous DNA-AgNCs. (f) Colony-forming units (CFUs) of *S. aureus* when treated with fibrous DNA-AgNCs at 0.5 μM DNA, analyzed 6 h post-treatment. Error bars represent mean ± SEM, *n* = 3, *P < 0.05. Significant reduction from the 1HP is denoted with daggers via one-way ANOVA.

Following physical characterization, the antibacterial properties of fibrous DNA-AgNCs were tested against planktonic *S. aureus* (Figure 4f) demonstrating stronger bactericidal activity compared to that of HP DNA-AgNCs. No significant differences in activity were observed among the various fibrous DNA-AgNCs. Thus, incorporating multiple hairpins on a single monomer was not advantageous over a single hairpin per monomer. Similarly, altering flexibility did not further enhance antibacterial efficacy.

### Fibrous DNA-AgNCs Restrict Intracellular *S. aureus* in Resident Bone Cells

It is now documented that resident bone cells, including bone-forming osteoblasts, serve as reservoirs of viable *S. aureus*. In the absence of a delivery reagent, DNA-AgNCs remain extracellular. As such, DNA-AgNCs were complexed with Lipofectamine 2000 (L2K) to deliver nanostructures to primary murine osteoblasts. The 1HP DNA-AgNCs and fibrous DNA-AgNCs were successfully delivered intracellularly to osteoblasts and visualized via fluorescent microscopy (Figure 5 and supporting Information Figure S6). Importantly, the delivery of all DNA-AgNCs to *S. aureus*-infected osteoblasts significantly reduced intracellular bacterial burden. Our results indicate that all tested DNA-AgNCs reduced *S. aureus* intracellular CFU counts compared to untreated and carrier-only controls, with the 2HP-2T and 2HP-3T fibrous DNA-AgNCs demonstrating the greatest efficacy. Although these constructs did induce an inflammatory response in osteoblasts in IL-6, we did not observe any off-target toxicity, and our data suggest that inflammatory responses are independent of TLR-9 activation of the NF- κB pathway (Figure S5). Importantly, at the concentration tested in primary murine osteoblasts, we were able to significantly reduce bacterial burden in the absence of any undesired mammalian cell death.

**Figure 5:**
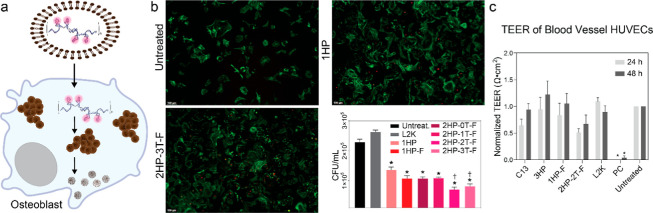
(a) Primary murine osteoblasts were first infected with *S. aureus* (MOI 75:1), then transfected with DNA-AgNCs (at 0.5 μM DNA) using Lipofectamine 2000 (L2K). (b) Uptake images of osteoblasts at 2 h post-transfection with DNA-AgNCs (more images can be found in Figure S6) and CFU of *S. aureus* at 6 h-post infection for various DNA-AgNCs. Error bars represent mean ± SEM, *n* = 3, *P < 0.05. Daggers denote significant reduction in CFU when compared to 1HP at 0.5 μM DNA. (c) TEER values for 1HP, 3HP, 1HP-F, and 2HP-2T-F DNA-AgNCs. Error bars represent mean ± SEM, *n* = 3, *P < 0.05.

### DNA-AgNCs Do Not Disrupt Membrane Integrity of Blood Vessel Human Umbilical Vein Endothelial Cells

The transepithelial electrical resistance (TEER) values measure the barrier integrity of the HUVEC tubules. TEER measurements can be used to assess the toxicity of treatment prior to in vivo studies, giving a better understanding of the system after mammalian cell culture work. From this data, we show that at bactericidal concentrations, DNA-AgNCs are not damaging to the barrier integrity in blood vessel HUVECs over 48 h when delivered with L2K (Figure 5c).

### DNA-AgNCs Produce Reactive Oxygen Species

Silver nanoparticles kill bacteria through multiple mechanisms, including through the formation of reactive oxygen species (ROS).^66,67^ While the mechanism of DNA-AgNCs has not been studied completely, it is hypothesized that DNA-AgNCs kill bacteria in a similar manner. To verify the ability of 1HP DNA-AgNCs to generate ROS, electron paramagnetic resonance (EPR) spectroscopy was employed using 1-hydroxy-3-carboxy-2,2,5,5-tetramethylpyrrolidine (CPH) as a spin-probe molecule. CPH readily reacts with ROS and free radicals to form a stable nitroxide radical, producing a characteristic three-line EPR spectrum. Upon illumination with 554 nm light, 1HP DNA-AgNCs are expected to undergo electronic excitation from the ground state to an excited singlet state followed by intersystem crossing to the triplet state. The latter can transfer energy or electrons to surrounding oxygen molecules, thereby promoting the formation of ROS. Consistent with our expectations, we observed a pronounced increase in the EPR signal intensity after light exposure (Figure 6a). The irradiated sample exhibited a well-resolved triplet pattern typical of the CPH radical, confirming its interaction with generated ROS. After 2 min of irradiation, the signal intensity reached approximately 5.0 × 10^5^ counts, compared to 1.6 × 10^5^ counts in the nonirradiated control (1HP DNA-AgNCs + CPH in buffer) and near-baseline levels (1.8 × 10^4^ counts) in buffer-only samples. These results provide direct evidence that 1HP DNA-AgNCs actively facilitate the generation of reactive oxygen species under light irradiation, consistent with a photoinduced ROS formation mechanism.

**Figure 6:**
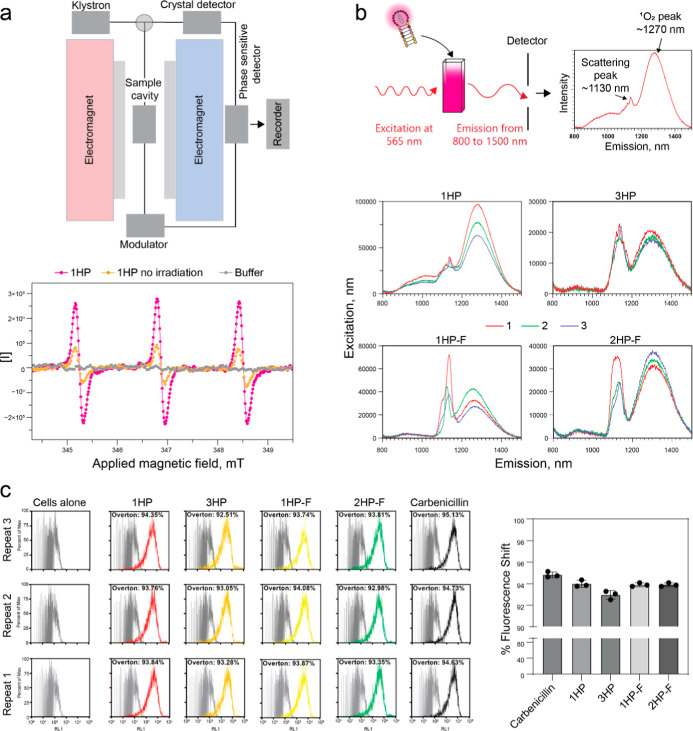
(a) Experimental workflow of electron paramagnetic resonance (EPR) spectroscopy of 1HP DNA-AgNCs and EPR results for samples irradiated with 554 nm light (pink), nonirradiated samples (orange), and buffer alone (gray). (b) Evaluating singlet oxygen production using fluorescence emission spectroscopy when excited with 565 nm light. Each construct had 3 emission spectra taken consecutively with red lines as the first, green lines as the second, and blue lines as the third read. (c) *S. aureus* was untreated (cells alone), treated with DNA-AgNCs (4 μM DNA), or treated with carbenicillin (50 μg/mL) for 3 h. Following the treatment, bacteria were stained with the LIVE/DEAD BacLight Bacterial Viability Kit and evaluated with flow cytometry. Representative fluorescence intensity histograms from *n* = 3 experiments are shown. The percentage of dead cells is denoted with the Overton percentage relative to the untreated control.

To confirm the presence of ROS, particularly singlet oxygen (^1^O_2_), we used fluorescence spectroscopy (Figure 6b). Samples were excited at 565 nm, and the emission spectra were measured in the 800–1500 nm range. Each spectrum was expected to show two intense peaks: one at approximately 1130 nm corresponding to the scattering peak (twice the excitation wavelength) and another peak at 1270 nm, characteristic of ^1^O_2_ emission. From these data, we saw that all samples had some level of ^1^O_2_ production, with 1HP DNA-AgNCs producing the highest amount of ^1^O_2_. Furthermore, each sample was measured three times consecutively, during which a gradual decrease in the intensity of the ^1^O_2_ peak was noted. This reduction may result from absorption of ^1^O_2_ by the samples or from sample degradation caused by the environmental stress associated with ^1^O_2_ exposure. Among the tested samples, 1HP produced the highest amount of ^1^O_2_, while 3HP produced the lowest amount, with the fiber samples falling in between. These results show that although all samples produce some ^1^O_2_, and DNA-AgNCs likely exert antibacterial activity through additional mechanisms not solely through ROS generation, similar to the behavior observed for silver nanoparticles.

To assess the impact of DNA-AgNCs on bacterial membrane integrity, cells were stained with propidium iodide, which penetrates only cells with compromised membranes. Treated cells exhibited a significant increase in fluorescence, as reflected by a peak shift. Across all treated sample types, flow cytometry quantified over 93% of cells showing this shift (Figure 6c), indicating that DNA-AgNCs disrupt bacterial membranes and induce cell death. Since cell viability experiments were performed at ambient light conditions, we should expect some singlet oxygen generation. To directly measure this effect, we compared the cell viability when treated and excited with bright white light to that on treatment plates that remained in the dark. While there was not a significant difference between the “high light” and “low light (dark)” conditions, there was a decrease in bacteria cell growth for the 3HP and 2HP-F samples tested, indicating that there could be ROS production occurring, as well as other mechanisms that kill bacteria (Figure S7). This needs to be evaluated in future studies.

### DNA-AgNCs Have Continued Antibacterial Efficacy at Different Storage Conditions

As therapeutic nucleic acids progress to clinical use, the need for cold chain transportation remains a problem; furthermore, the freeze–thaw cycles break down nucleic acids and lowers the quality of potential therapeutics.^68–70^ Since the ideal storage of therapeutics would be at room temperature (RT), we evaluated the efficacy over time for DNA-AgNCs at these storage conditions. Multiple DNA-AgNCs were evaluated for their stability and retention of antibacterial properties over the course of 4 weeks with storage at *RT* or at 4 °C. The 1HP construct lost most of its fluorescence within 1 week, while all other samples retained detectable fluorescence for at least 3 weeks. The 3HP construct remained pink with the fluorescence diminishing more slowly than for the 1HP sample. Interestingly, the color shifted for each of the fibrous structures, from red-orange to orange-yellow, suggesting that higher-order structures can protect the fluorescence stability over time (Figure 7). This trend was consistent for samples kept at both *RT* and 4 °C. The EEMs also show the change in fluorescence for the fiber structures, as the maximum of the fluorescence peak is shifting from 600 to 700 nm to 500–600 nm. Importantly, the bacterial efficacy was not observed to have a significant change between weeks 1 through 4. The samples had consistent antibacterial efficacy over the 4 week storage period under both conditions. One notable exception was the 1HP sample at 3 weeks of storage where the sample stored at RT performed better than the sample stored at 4 °C.

**Figure 7:**
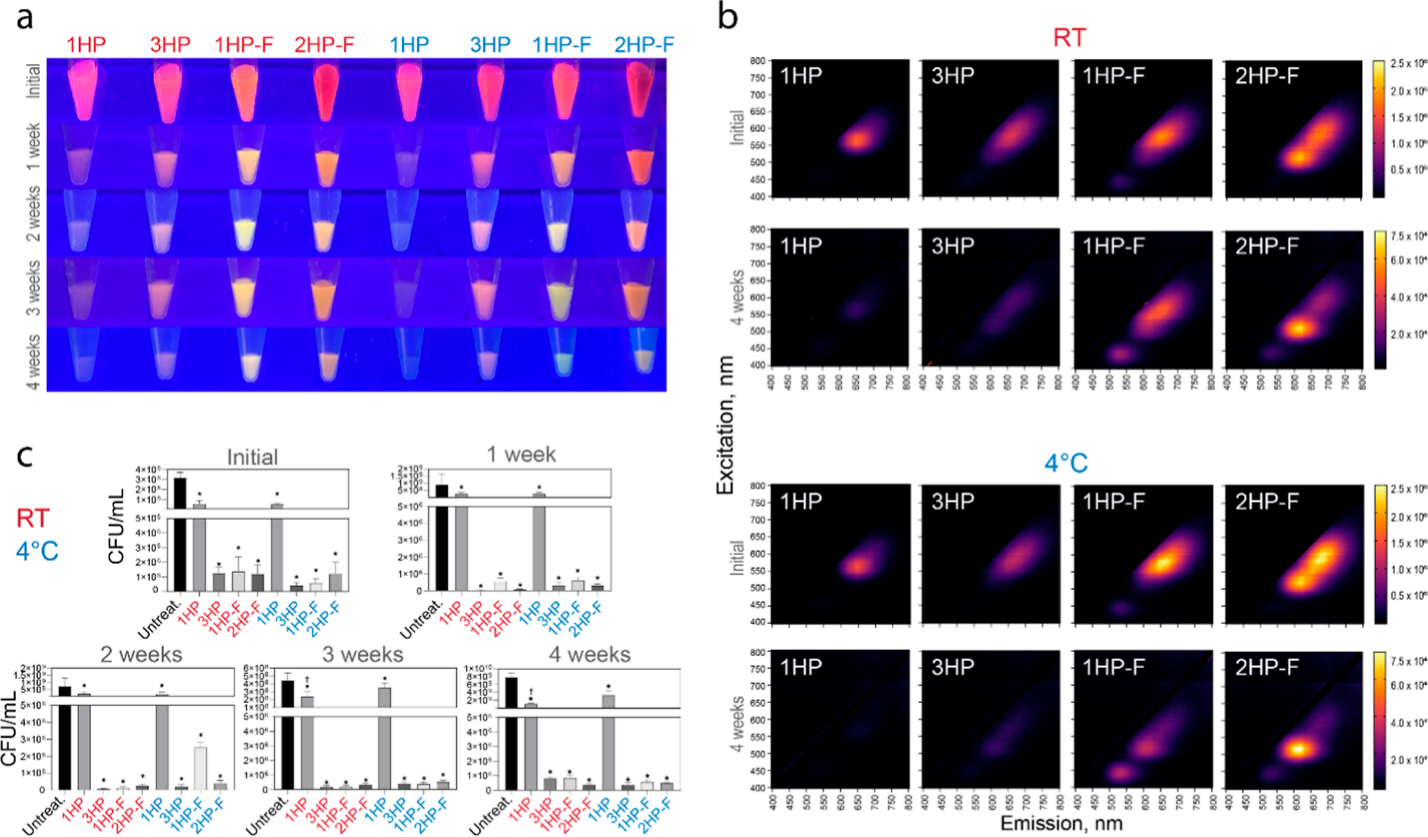
Evaluation of fluorescence and antibacterial properties of DNA-AgNCs over 4 weeks. (a) Images of 1HP, 3HP, 1HP-F, and 2HP-F stored at room temperature (*RT*) or at 4 °C over 4 weeks. (b) Excitation–emission spectra of 1HP, 3HP, 1HP-F, and 2HP-F DNA-AgNCs stored at room temperature (*RT*) or at 4 °C, evaluating the difference in fluorescence initially to 4 weeks post synthesis. (c) The antibacterial efficacy of 1HP, 3HP, 1HP-F, and 2HP-F DNA-AgNCs initial to 4 weeks post synthesis against *S. aureus* at 4 μM DNA. Error bars represent mean ± SEM, *n* = 3, *P < 0.05.

## Conclusion

In conclusion, DNA-AgNCs hold significant promise as antibacterial therapeutics, with additional fluorescence probing capabilities. Since DNA sequence and structure can be precisely tuned, DNA-AgNCs can be engineered for specific purposes, thereby optimizing stability, fluorescence properties, and antimicrobial activity. Our work demonstrates that C13–3HP DNA-AgNCs are effective antimicrobials against antibiotic-resistant pathogens. Specifically, we show that the C13 hairpin has enhanced stability, while possessing optimal fluorescence for fluorescence probing. Importantly, C13-HP exhibits antimicrobial activity against clinically relevant Gram-negative and Gram-positive bacterial strains that display antibiotic resistance. The fibrous DNA-AgNCs containing multiple C13-HPs increase the delivery concentration of silver ions, further enhancing the antimicrobial activity. Importantly, C13-HPs and fibrous DNA-AgNCs can be effective in reducing planktonic bacterial burden and can additionally be complexed with a lipid barrier to significantly restrict intracellular burden in resident bone cells. However, intracellular use will require careful dose optimization to minimize cytotoxicity and possible immune stimulation. Moreover, DNA-AgNCs act as effective antimicrobials over the course of 4 weeks with minimal changes in activity. Collectively, these results establish the therapeutic potential of the highly modular bifunctional DNA-AgNC platform as alternatives to antibiotics, recognizing the need for further experimental studies to investigate the mechanisms underlying the antimicrobial activity of DNA-AgNCs and assessing their efficiency in vivo.

## Materials and Methods

### DNA-AgNCs C5–15 Hairpin Synthesis

To create each unique DNA-AgNC, AgNO_3_ was mixed with the specified DNA template, which yielded a final DNA concentration of 25 μM, in 4 mM NH_4_OAc, with a pH of 6.9, providing a 1:1 ratio of Ag^+^:Cytosine. Each sample solution was vortexed, centrifuged, and heated to 95 °C for 2 min. Then, the samples were immediately incubated in an ice bath for 20 min. During each incubation period, a fresh 10 mM NaBH_4_ solution was made for the individual DNA-AgNC syntheses by using cold water. To reduce Ag^+^, after incubation, an equimolar amount of NaBH_4_ was added to each sample, mixed by pipetting and quick centrifugation, and allowed to sit at 4 °C for a minimum of 15 h. Then, excess reagents were removed by washing the newly formed DNA-AgNCs in a 3 kDa molecular weight cutoff centrifuge filter with 20% initial volume of 4 mM NH_4_OAc, and spun 3 times at 12,000 RCF for 25 min at 4 °C. Estimated concentrations of the pure DNA-AgNC solutions were quantified by measuring the absorption of 260 nm light using Nanodrop 2000 and diluted to the concentration of interest. All DNA sequences are reported in the Supporting Information, Table S1.

### Fibrous DNA-AgNCs

Equimolar amounts of complementary fiber strands A and B were incubated separately at 95 °C for 5 min. After strand incubation, the complementary strands were added to AgNO_3_ and 20 mM NH_4_Oac (final concentration of 4 mM NH_4_OAc), then incubated at 25 °C for 20 min. During each incubation period, a fresh 10 mM NaBH_4_ solution, using cold, double-deionized water, was made. To reduce Ag^+^, after incubation, an equimolar amount of NaBH_4_ was added to each sample, mixed by pipetting, and allowed to sit at 4 °C for a minimum of 15 h. Fiber samples were washed by following the hairpin washing protocol. The melting temperature (36 °C) of the strands and 4 °C was also evaluated to find the best temperature for synthesis. 25 °C was determined to be the best temperature for the second incubation because the DNA-AgNC run on 8% native polyacrylamide gel electrophoresis was the most similar to that of the DNA fiber (Figure S4).

### Energy Dispersive X-ray Spectroscopy Measurements

C5–15 DNA-AgNCs were prepared at a 10 μM concentration. Silica wafers were attached to the SEM pegs by using copper tape to allow for the electrons to transfer off the sample. Each sample was added to the silica wafer in 5 μL droplets and then dried at 45 °C. Once the droplet was dry, a second droplet was added on top of the initial spot to increase the width of the sample. This was repeated until a total of 20 μL was added. From there, desktop SEM (Thermo-Fisher Phenom XL desktop SEM) was used to perform EDS. The percentage of all atoms was found. Based on the known number of phosphates per hairpin, the number of silver atoms was calculated. This was completed in triplicate, with the average and standard deviation calculated.

### AFM Imaging and Image Analysis

Samples for AFM imaging were prepared by depositing DNA structures onto freshly cleaved mica modified with 1-(3- aminoropyl) silatrane (APS) according to the previously described protocol.^71,72^ Briefly, 5 μL of DNA solutions at various concentrations were deposited onto the modified mica and incubated for 2 min. Unbound nucleic acids and excess salts were removed by washing the surface twice with 50 μL of deionized water, after which the mica was dried under a gentle stream of argon gas. AFM imaging was performed using a MultiMode AFM Nanoscope V system (Bruker Instruments, Santa Barbara, CA) operated in PeakForce tapping mode with MSNL-E or MSNL-F probes from Bruker. PeakForce parameters, including amplitude, frequency, and set point, were optimized to maintain peak interaction forces below 70 pN providing high-resolution imaging. AFM images were processed and analyzed using the open-source software package Gwyddion. ImageJ software equipped with the Skeleton plugin was used to extract and quantify the lengths of the formed fibrous structures.

### Building Atomic Models of C13–Ag_10_ HP Structures

The stem region of DNA was built by the Accelrys Discovery Studio. An initial hairpin (HP) loop structure was built based on an NMR DNA HP loop (PDB ID: 1JVE).^73^ The first base in the HP loop was replaced by cytosine using Accelrys Discovery Studio. The second to the last base was replaced by 12 cytosine bases associated with Ag_10_ cluster, whose atomic conformation was reported in our previous study.^74^ Energy minimization was applied to the assembled C13–Ag_10_ structures to refine the atomic geometry using CHARMM36 nucleic acids force fields and Ag atom force fields.^75^ The energy-minimized C13–Ag_10_ structure was used to build C13–Ag_10_ structures and C13–Ag_10_ fibers. The energy minimizations were applied to the C13–Ag_10_ structures and C13–Ag_10_ fibers.

### Fluorescence Measurements

Using a 96-well plate, 100 μL of each sample at a concentration of 10 μM was pipetted into each well. The plate was loaded into a Tecan Spark microplate reader, and the 3D excitation–emission matrices were recorded. The excitation data were measured over a range of 350–700 nm with a manual gain of 150 nm and a step size of 5 nm between measurements. The emission data were also measured with a manual gain of 150 but over a range of 400–850 nm with a 5 nm step size between measurements. Once all the data were recorded, it was plotted using MagicPlot Pro. These measurements were taken weekly or biweekly for 4 weeks to evaluate the change in fluorescence over time.

### Determination of Minimum Inhibitory Concentration

3HP DNA-AgNCs were tested against American Type Culture Collection (ATCC) standard strains (*E. coli* 25922, *P. aeruginosa* 27853, *K. pneumoniae* 13883, *S. aureus* 29213, and *E. faecalis* 29212). The resistant bacterial isolates belonged to the Bacteriology Laboratory LIM49 strain collection and were isolated from bloodstream infections of Hospital das Clínicas, Faculdade de Medicina, Universidade de São Paulo, São Paulo. The multiresistant strains tested were *A. baumannii*, *P. aeruginosa*, *K. pneumoniae*, *S. aureus* (MRSA), and *E. faecalis* (VRE). Each strain was cultivated aerobically on blood agar plates at 37 °C for 24 h to perform the susceptibility tests. MIC was determined by broth microdilution test using Mueller–Hinton Broth -MHB II (Difco, BD, USA) for the 3HP DNA-AgNCs according to Clinical and Laboratory Standards Institute (CLSI) for antimicrobial susceptibility testing.^76^ The MIC of 3HP DNA-AgNCs was determined against five ATCC reference strains (EC 25922, PA 27853, KP 13883, SA 29213, and EF 29212) in duplicate and in three independent experiments. The 96-well assay plates containing different concentrations of 3HP DNA-AgNCs ranged from 8 to 0.0025 μM and were obtained by 2-fold serial dilution and incubated at 35 ± 2 °C for 16 to 18 h, and the presence of turbidity on each concentration was visually determined with observation under transmitted light. Negative control was inoculated under identical conditions but without the addition of 3HP DNA-AgNCs, and sterility control was performed with MHB II without bacterial inoculation. The results were read by visually evaluating the turbidity of each well. The first well with no turbidity was defined as the MIC, expressed in μM. We also performed the same experiment with only the 3HP DNA-AgNCs diluent and determined that it did not interfere with the MICs, as all bacteria grew in all the diluent concentrations tested (Figures S2 and S3).

### Determination of Minimum Bactericidal Concentration

To determine the MBC, 10 μL of each well showing no obvious bacterial growth on the MIC test was seeded onto blood agar plates by streaking and incubated at 37 °C for 24 h. Subsequently, the plates were examined, and the lowest concentration at which no visible growth was observed was taken as the MBC of the 3HP DNA-AgNCs.

### Mammalian Cell Viability Assays

To evaluate cell viability after transfection, an MTS assay was conducted using a 96-well flat-bottom Greiner plate. A total of 20 μL of MTS reagent was added to 100 μL of cells per well. The absorbance was measured at a wavelength of 638 nm by using a Tecan Spark plate reader. Each condition was tested in biological triplicates, and the results were averaged and normalized to the cell-only control for analysis.

### *S. aureus* Propagation

*S. aureus* strain UAMS-1 was grown on Luria broth (LB) agar plates overnight, followed by incubation in LB broth at 37 °C and 5% CO2 overnight. The number of colony-forming units (CFUs) was determined by using a Genespec3 spectrophotometer as previously described (MiraiBio Inc.).

### Bacterial Viability

*S. aureus* was seeded at a density of 1 × 10^6^ CFUs per well in a 96-well plate. Cells were left untreated or treated with the indicated concentrations of DNA-AgNCs in LB broth for 6 h on an orbital shaker at 37 °C and 5% CO_2_. At 6 h, post incubation, serial dilutions were performed, and CFUs were plated on LB agar plates overnight. The number of viable colonies was assessed by colony counting. This time point was chosen based on the prior studies that demonstrated changes in intracellular bacterial burden in osteoblasts between 6 and 8 h.^77,78^ For light/dark experiments, *S. aureus* was seeded at a density of 1 × 10^6^ CFUs per well in a 96-well plate. Cells were left untreated or treated with 4 μM DNA-AgNCs in LB broth for 6 h. For light treatments, the treatment plate was hit with bright white light for 3 h and ambient light for 3 h. For dark treatments, the treatment plate was left in the dark and covered with aluminum foil. At 6 h post incubation, serial dilutions were performed, and CFUs were plated on LB agar plates overnight. The number of viable colonies was assessed by colony counting.

### Measurements of Reactive Oxygen Species Using Electron Paramagnetic Resonance

Electron Paramagnetic Resonance (EPR) spectroscopy was used to investigate the generation of reactive species by 1HP in the presence of a spin trap. The spin trap, 1-hydroxy-3-carboxy-2,2,5,5-tetramethylpyrrolidine (CPH), served as a sensitive probe for detecting reactive oxygen species (ROS) and free radicals. Three experimental conditions were compared: (1) only buffer; (2) dark control, a mixture containing 1HP (10 μM) aged for 24 h and CPH (200 μM) was maintained in darkness, and care was taken to minimize any incidental light exposure during measurement; and (3) light-irradiated sample, an identical solution containing 1HP (10 μM) and CPH (200 μM) was illuminated with continuous mint LED light (Thorlabs, λ_max_ = 554 nm) for 2 min prior to EPR analysis. The LED intensity was regulated in constant-current mode at 1 A using a four-channel LED Driver (DC4100, Thorlabs, Inc.). EPR spectra were acquired on a Bruker E-Scan spectrometer.

### Measuring Singlet Oxygen Production

Samples were prepared with 26 μM silver, varying concentrations of DNA, for 4 constructs: 1HP, 3HP, 1HP-Fiber, and 2HP-0T-Fiber. Using a Horiba Scientific FluoroMax+, the samples were irradiated at 565 nm light, and the emission spectra was gathered from 800 to 1500 nm to capture both the scattering peak at 1130 nm and the singlet oxygen (^1^O_2_) peak at 1270 nm. Data were plotted using GraphPad Prism.

### Evaluating Membrane Disruption of DNA-AgNCs Against *S. aureus*

*S. aureus* was seeded at a density of 1 × 10^6^ CFUs per well in a 96-well plate. Cells were left untreated or treated with 4 μM DNA-AgNCs in LB broth for 3 h at 37 °C. After 3 h, each well was centrifuged at 300 xg for 5 min to pellet the cells. Supernatant was removed and replaced with PBS +1.5 μL/mL SYTO9 and 1.5 μL/mL Propidium Iodide, following the LIVE/DEAD BacLight Bacterial Viability Kit for microscopy and quantitative assays from ThermoFisher Scientific. The cells were incubated in the dark at room temperature for 30 min to allow for proper staining and analyzed through flow cytometry (AttuneNXT Acoustic Focusing Cytometer, ThermoFisher Scientific) to assess membrane permeability. SYTO9 stains all cells, whereas propidium iodide stains only dead cells with permeable membranes.^79^ The percentage of dead cells was quantified as propidium iodide positive events using the Overton percentage given relative to the cells alone control.

### Evaluating Mammalian Cell Toxicity and Immunostimulation

HEK-Blue hTLR9 cells were cultured according to InvivoGen’s protocols at 37 °C and 5% CO_2_ in DMEM supplemented with 10% heat-inactivated FBS, 1% penicillin–streptomycin, 100 μg/mL Normocin, 100 μg/mL Zeocin, and 10 μg/mL Blasticidin. For reverse transfection in 96-well plates, ∼80,000 cells per well were seeded onto preloaded treatments or controls. Poly I/C (5 μg/mL) was used as a positive control for SEAP activation in the Quanti-Blue assay. Intracellular delivery of DNA-AgNC treatments (4 μM) was achieved by precomplexing DNA-AgNCs with L2K at room temperature for 30 min before transfection. After 24 h, SEAP activation and cell viability were assessed following the manufacturer’s protocols. For SEAP detection, 20 μL of cell supernatant was incubated for 2 h with 180 μL of Quanti-Blue solution, and absorbance was measured at 638 nm using a Tecan Spark plate reader. Cell viability was evaluated using an MTS colorimetric assay, performed by adding a reagent equivalent to 20% of the final well volume, followed by a 2 h incubation with absorbance recorded at 490 nm. All assays were performed in biological triplicate. Results were averaged, normalized to untreated (cell-only) controls, and expressed as fold induction. Significance was calculated in GraphPad Prism by using a two-way ANOVA.

### Isolation and Maintenance of Primary Bone Cells

Whole calvaria were harvested from 2 to 3 day-old neonatal mice and differentiated for 10 days, as previously described.^80–84^ Differentiation was validated by alkaline phosphatase staining using a commercial kit (Abcam) and confirmed by microscopy, following established protocols.^84^

### Immunofluorescence Microscopy of Transfected Osteoblasts

Osteoblasts were transfected with DNA-AgNCs by using Lipofectamine 2000. Cells were fixed at 2 h post-transfection and processed for immunofluorescence microscopy for actin using Alexa Fluor 488 Phalloidin (ThermoFisher Scientific, A12379, 100 nM). Cells were imaged using a Leica DMi8 fluorescence microscope.

### Bacterial Infection of Osteoblasts

Primary murine osteoblasts were seeded at a density of 1 × 10^6^ cells per well and infected with *S. aureus* at a multiplicity of infection (MOI) of 75 bacteria per host cell in antibiotic-free media for 2 h. Following infection, the medium was replaced with fresh medium containing 1% penicillin–streptomycin. At 8 h post infection, cell supernatants were collected for analysis. Additionally, osteoblasts were lysed using Saponin, and intracellular CFUs were plated on LB agar plates overnight. The number of viable colonies was assessed by colony counting. This time point was chosen based on prior studies that demonstrated changes in intracellular bacterial burden in osteoblasts between 6 and 8 h.^77,78^

### DNA-AgNCs Transfection of Osteoblasts

Primary murine osteoblasts were seeded at a density of 1 × 10^6^ cells per well and infected with *S. aureus,* as previously described. Following infection, the cells were transfected with 0.5 μM DNA-AgNCs using the lipid-based carrier, L2K (Invitrogen), in antibiotic-free medium for 2 h. Following transfection, fresh medium containing 1% penicillin–streptomycin was added. At 8 h post infection, cell supernatants were collected for analysis. Additionally, osteoblasts were lysed using Saponin, and intracellular CFUs were plated on LB agar plates overnight. The number of viable colonies was assessed by colony counting.

### Enzyme-Linked Immunosorbent Assays

Specific capture enzyme-linked immunosorbent assays (ELISAs) were performed to measure the osteoblast production of interleukin-6 (IL-6) in response to *S. aureus* infection and transfection of nanoparticles. IL-6 production was determined using commercially available antibody pairs (BD Biosciences; 554400, 554402). Recombinant proteins for each target were used to generate standard curves, and protein concentrations were determined by matching sample absorbance values to the corresponding standard curve.

### Transepithelial Electrical Resistance Measurements

OrganoReady Blood Vessel HUVEC (Mimetas BV, The Netherlands) was used to assess toxicity on day 6 of culture. Samples were used with or without complexation with Lipofectamine 2000 (ThermoFisher) at room temperature for 30 min prior to bringing up to the final volume in OrganoMedium HUVEC-ABM. The final concentrations of AgNCs tested were 0.5 μM with L2K and were compared to L2K alone and staurosporine (100 nM) as a positive control. Media were refreshed every other day up until the assay window. Prior to treatment, transendothelial electrical resistance (OrganoTEER, Mimetas BV, The Netherlands) was measured as a baseline. Samples were added to the apical side of the HUVEC tubules and were maintained on an OrganoFlow at 7°/8 min settings for continuous gravity-driven perfusion within a 37 °C, 5% CO_2_ incubator. TEER was measured at 24 and 48 h post-treatment following 30 min equilibration at RT.

### DNA-AgNCs Aging Studies

A large stock of DNA-AgNCs were synthesized following the protocols above and kept at either 4 °C or room temperature (*RT*, 25 °C) for up to 4 weeks post synthesis. The day after synthesis, the entire stock of DNA-AgNCs was washed, following the protocol above. All samples were kept in the dark until use. From there, the samples were taken from the stock each week and used against UAMS-1 *S. aureus*, following the bacterial viability protocol, and the photochemical properties were evaluated using the fluorescence measurements protocol. EEM was plotted using GraphPad Prism.

### Statistical Analysis

All experiments were performed in at least three independent replicates, with exact numbers indicated in the figure legends. Data are presented as mean ± SEM. Statistical comparisons between groups were performed using GraphPad Prism, and *p*-values <0.05 were considered statistically significant.

## Supplementary Material



## Data Availability

The data supporting the findings of this study are available from the corresponding author upon reasonable request.

## Notes

DNA sequences used in this study, additional figures for experiments described in the text, and MIC and MBC values of tested compounds (PDF)
