# Supplementary material for: Spatially Organized DNA-Templated Silver Nanoclusters as Potent Antimicrobial Agents for ESKAPE Infections
Source: ACS Appl Mater Interfaces. 2026 Mar 16;18(12):17527–41. doi: 10.1021/acsami.5c25898 (PMC13049360; doi:10.1021/acsami.5c25898)
Supplement: Supplementary file 1 [file am5c25898_si_001.pdf]

Supporting Information for

Spatially Organized DNA-templated Silver Nanoclusters as Potent Antimicrobial Agents for ESKAPE Infections

Elizabeth Skelly[1], Krishna Majithia[2], Laura P. Rebolledo[1], Camila Fonseca Rizek[3], Silvia Figueiredo Costa[3,7], Alora R. Dunnavant[1], Cheyenne Vasquez[1], Alexander J. Lushnikov[4], Alexey V. Krasnoslobodtsev[4], Taejin Kim[5], Morgan R. Chandler[1,8], Renata de Freitas Saito[6], Roger Chammas[6], M. Brittany Johnson[2], Kirill A. Afonin[1]*

[1]Chemistry and Nanoscale Science Program, Department of Chemistry, University of North Carolina at Charlotte, Charlotte, NC, 28223, USA
[2]Department of Biology, University of North Carolina at Charlotte, Charlotte, NC, 28223, USA
[3]Laboratório de Investigação Médica 49, Departamento de Infectologia e Medicina Tropical da Faculdade de Medicina da Universidade de São Paulo, Av. Dr. Eneas de Carvalho Aguiar, 470, São Paulo, Brazil
[4]Department of Physics, University of Nebraska Omaha, Omaha, NE, 68182, USA
[5]Department of Physical Sciences, West Virginia University Institute of Technology, Beckley, WV, 25801, USA
[6]Centro de Investigação Translacional em Oncologia (LIM24), Departamento de Radiologia e Oncologia, Faculdade de Medicina da Universidade de São Paulo and Instituto do Câncer do Estado de São Paulo, São Paulo, SP, Brazil
[7]Centres for Antimicrobial Optimisation Network (CAMO-Net) Brazil, Faculty of Medicine, University of São Paulo, São Paulo, Brazil
[8]MIMETAS US, Inc, Gaithersburg, MD, 20878, USA

*- correspondence to kafonin@charlotte.edu

Table S1: Sequences used in this project

Complex Type	Strand Name	Sequence (5'—3')
Single HP	C5	TATCCGTCCCCCACGGATA
Single HP	C6	TATCCGTCCCCCCACGGATA
Single HP	C7	TATCCGTCCCCCCCACGGATA
Single HP	C8	TATCCGTCCCCCCCCACGGATA
Single HP	C9	TATCCGTCCCCCCCCCACGGATA
Single HP	C10	TATCCGTCCCCCCCCCCACGGATA
Single HP	C11	TATCCGTCCCCCCCCCCCACGGATA
Single HP	C12	TATCCGTCCCCCCCCCCCCACGGATA
Single HP	C13 (1HP)	TATCCGTCCCCCCCCCCCCCACGGATA
Single HP	C14	TATCCGTCCCCCCCCCCCCCCACGGATA
Single HP	C15	TATCCGTCCCCCCCCCCCCCCCACGGATA
Multiple HP	2HP	TATCCGTCCCCCCCCCCCCCACGGATATATCCGTCCCCCCCCCCCCCACGGATA
Multiple HP	3HP	TATCCGTCCCCCCCCCCCCCACGGATATATCCGTCCCCCCCCCCCCCACGGATAACGGATACCCCCCCCCCCCCTATCCGT
Multiple HP	3HP-1T	TATCCGTCCCCCCCCCCCCCACGGATATTATCCGTCCCCCCCCCCCCCACGGATATACGGATACCCCCCCCCCCCCTATCCGT
Multiple HP	3HP-2T	TATCCGTCCCCCCCCCCCCCACGGATATTTATCCGTCCCCCCCCCCCCCACGGATATTACGGATACCCCCCCCCCCCCTATCCGT
Multiple HP	3HP-3T	TATCCGTCCCCCCCCCCCCCACGGATATTTTATCCGTCCCCCCCCCCCCCACGGATATTTACGGATACCCCCCCCCCCCCTATCCGT
Fiber	1HP-F-A	GTTCATCTGCACCAACGGATACCCCCCCCCCCCCTATCCGTGGAATCCAAGGA
Fiber	1HP-F-B	TGGTGCAGATGAACACGGATACCCCCCCCCCCCCTATCCGTTCCTTGGATTCC
Fiber	2HP-0T-F-A	GTTCATCTGCACCAACGGATACCCCCCCCCCCCCTATCCGTTATCCGTCCCCCCCCCCCCCACGGATAGGAATCCAAGGA
Fiber	2HP-0T-F-B	TGGTGCAGATGAACACGGATACCCCCCCCCCCCCTATCCGTTATCCGTCCCCCCCCCCCCCACGGATATCCTTGGATTCC
Fiber	2HP-1T-F-A	GTTCATCTGCACCAACGGATACCCCCCCCCCCCCTATCCGTTTATCCGTCCCCCCCCCCCCCACGGATAGGAATCCAAGGA
Fiber	2HP-1T-F-B	TGGTGCAGATGAACACGGATACCCCCCCCCCCCCTATCCGTTTATCCGTCCCCCCCCCCCCCACGGATATCCTTGGATTCC
Fiber	2HP-2T-F-A	GTTCATCTGCACCAACGGATACCCCCCCCCCCCCTATCCGTTTTATCCGTCCCCCCCCCCCCCACGGATAGGAATCCAAGGA
Fiber	2HP-2T-F-B	TGGTGCAGATGAACACGGATACCCCCCCCCCCCCTATCCGTTTTATCCGTCCCCCCCCCCCCCACGGATATCCTTGGATTCC
Fiber	2HP-3T-F-A	GTTCATCTGCACCAACGGATACCCCCCCCCCCCCTATCCGTTTTTATCCGTCCCCCCCCCCCCCACGGATAGGAATCCAAGGA
Fiber	2HP-3T-F-B	TGGTGCAGATGAACACGGATACCCCCCCCCCCCCTATCCGTTTTTATCCGTCCCCCCCCCCCCCACGGATATCCTTGGATTCC

Cytosine-rich hairpin sections are underlined.

Supporting Figures

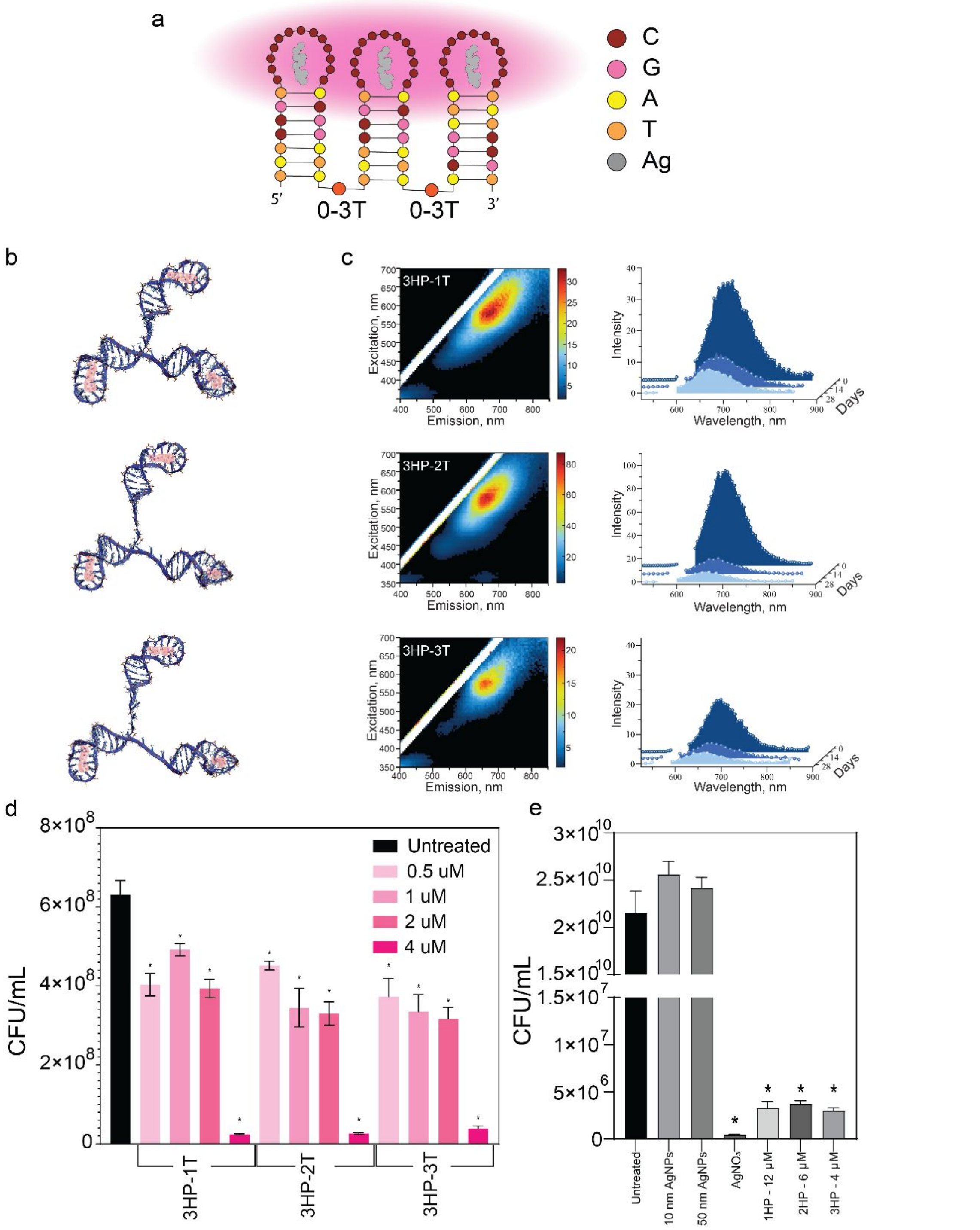

Figure S1. Evaluation of increased flexibility of 3HP DNA-AgNCs with additional thymines. (**a**) Depiction of the 3HP structure and sequence. (**b**) Computational modeling of the structure of 3HP with 1-3 additional thymines. (**c**) Initial 3D excitation-emission spectra with the change in intensity over 4 weeks. (**d**) The anti-bacterial efficacy of DNA-AgNCs at 0.5 to 4 μM. (**e**) Antibacterial effect of DNA-AgNCs as compared to silver nanoparticles (AgNPs at 120 μM) and silver nitrate ($AgNO_3$ at 120 μM). Error bars represent mean ± SEM, n = 3, *P < 0.05.

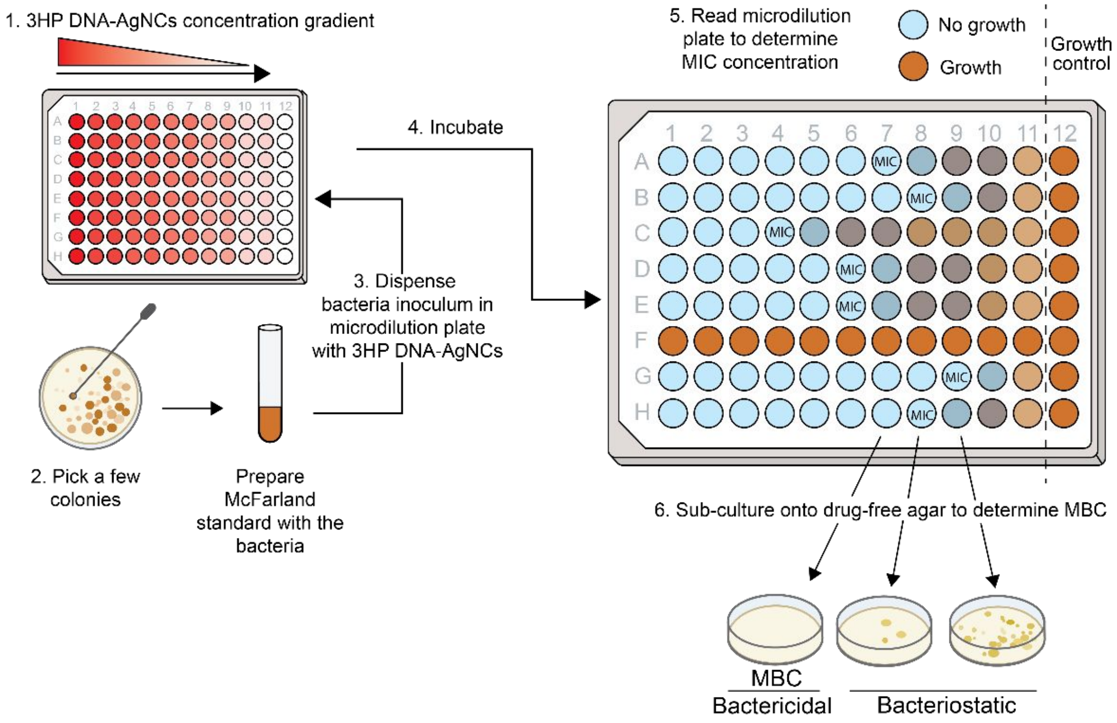

Figure S2. Scheme of MIC/MBC assay performed according to CLSI (Clinical and Laboratory Standards Institute) guidelines, (1) first prepare a serial dilution of C13-3HP DNA-AgNCs in a microdilution plate. (2) Prepare the inoculum by taking a few colonies from an agar plate with a sterile swab, prepare a 0.5 McFarland standard, and dilute the McFarland standard into the media. (3) Dispense the inoculum into the microdilution plate with the serial diluted C13-3HP DNA-AgNCs and incubate the plate at 37°C. (4) Read the plate to determine the MIC value. (5) Plate a portion of each well on an agar media, incubate and check for colonies to determine the MBC.

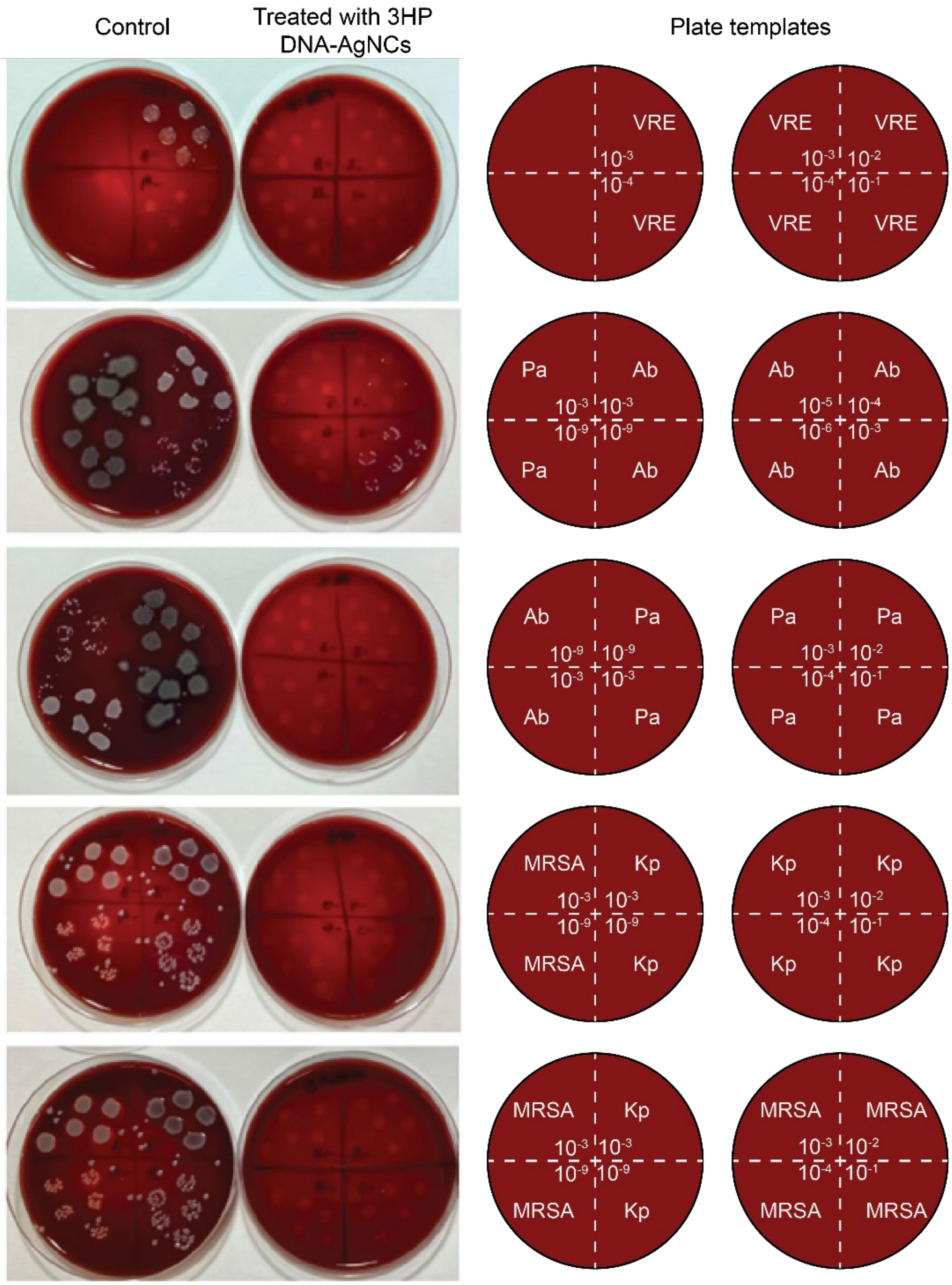

Figure S3. Measure the antimicrobial effects of 3HP DNA-AgNCs on antibiotic-resistant strains. After determining MIC, an aliquot was removed and up to 9 serial ten-fold dilutions were performed in Mueller–Hinton Broth to obtain 10^{-1} through 10^{-9} dilutions. An aliquot of 10 μl of each dilution was spotted five times onto a blood agar plate divided into four quadrants. After 24 hours of incubation at 37°C, colonies were observed. In the dilutions of control without 3HPs DNA-AgNCs, it was not possible to count single colonies, precluding the determination of CFU/mL. Legend: VRE (Vancomycin-resistant *Enterococcus faecalis*), Kp (Multidrug-resistant and Carbapenemic-resistant *Klebsiella pneumoniae*), Ab (Multidrug-resistant and Carbapenemic-resistant *Acinetobacter baumannii*), Pa (Multidrug-resistant *Pseudomonas aeruginosa*), and MRSA (Methicillin-resistant *Staphylococcus aureus*).

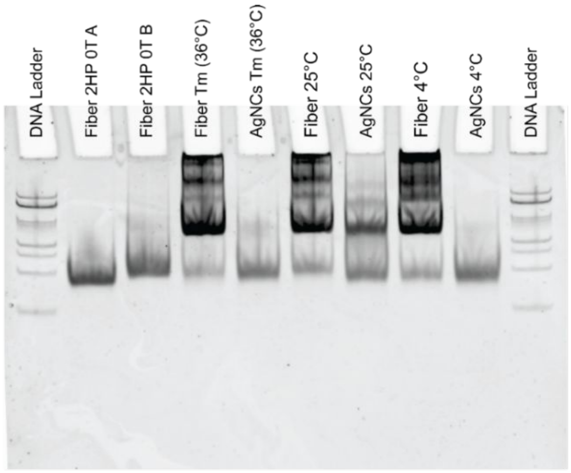

Figure S4. Evaluating Fiber DNA-AgNCs assembly incubation temperatures. Fiber DNA-AgNCs were found to have the most similar structure to Fiber DNA when incubated at 25°C, whereas the structures incubated at 36°C and 4°C look more similar to individual monomers. Thus, the Fiber DNA-AgNCs were synthesized with the incubation step at 25°C.

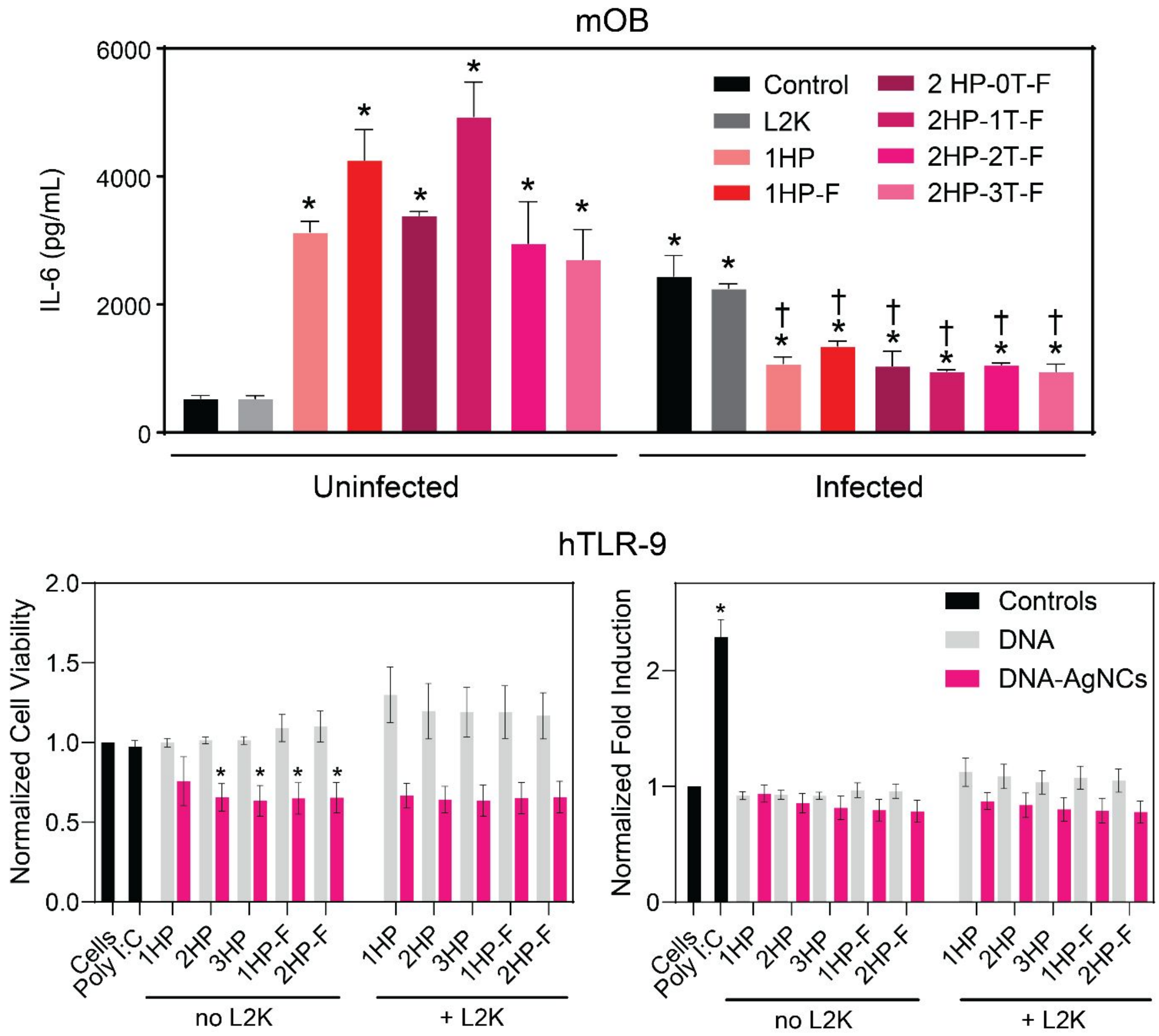

Figure S5. *Top*: Immunostimulation of primary murine osteoblasts following treatment with DNA-AgNCs at 0.5 μM in the absence and presence of *S. aureus* infection, 6 hours post-treatment. Asterisks denote significance from the treatment to cells alone. Daggers denote significance between the sample of interest with and without infection. *Bottom*: Mammalian cell toxicity of multiple hairpin DNA-AgNCs and the immunostimulatory properties of multiple hairpin structures against Human TLR9 Reporter HEK293 cells (hTLR9) at 4 μM DNA when treated with and without Lipofectamine 2000 to evaluate the stimulation of the NF-κB pathway 24 hours post-treatment. Asterisks denote significance from the treatment to cells alone.

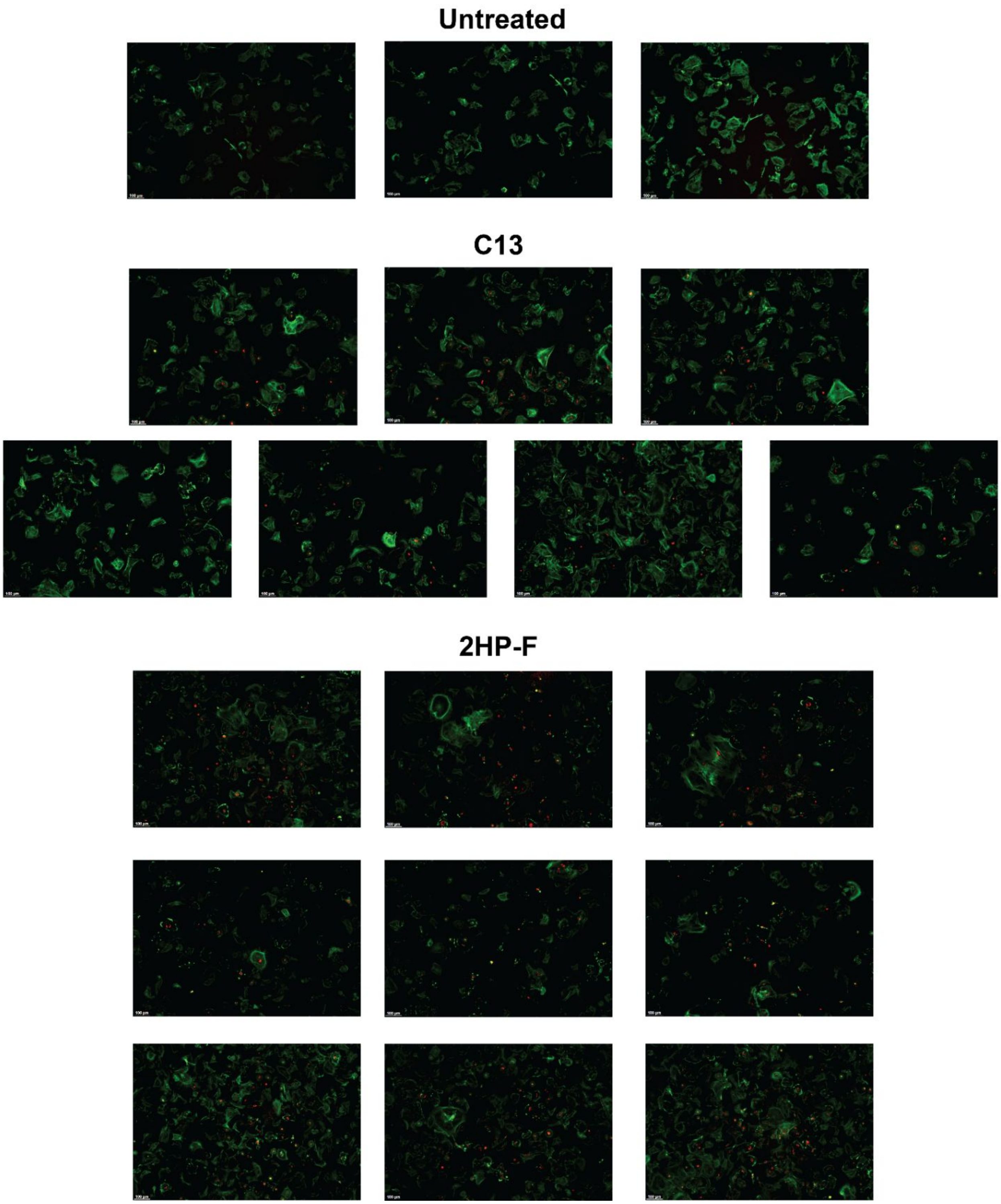

Figure S6. Uptake images of DNA-AgNCs when transfected into primary murine osteoblasts. From top to bottom: osteoblasts without transfected DNA-AgNCs, osteoblasts transfected with 2 μM C13 DNA-AgNCs, and osteoblasts transfected with 2 μM 2HP-F DNA-AgNCs.

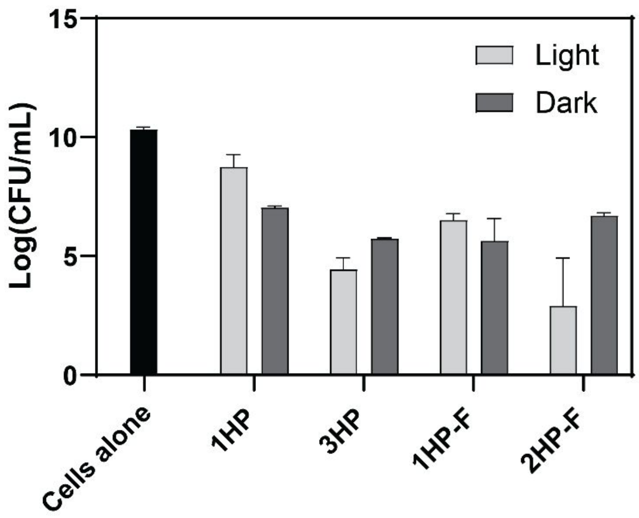

Figure S7. Evaluating *S. aureus* viability when treatments are excited with bright white light. Samples all showed significant cell death as compared to cells alone, however there was no significant difference between the light and dark samples.

Supporting Tables

SUSCEPTIBLE STRAINS

Microorganism	Gram	MIC (µM)	MBC (µM)	Resistent
Escherichia coli	negative	1 to 4	1 to 4	no
Pseudomona aeruginosa	negative	2 to 4	2 to 4	no
Klebsiella pneumoniae	negative	2 to 4	2 to 4	no
Staphylococcus aureus	positive	4	8	no
Enterococcus fecalis	positive	4	8	no

RESISTANT STRAINS

Microorganism	Gram	MIC (µM)	MBC (µM)	Resistent
Acinetobacter baumannii	negative	2	4	MDR, CARB-R
Pseudomonas aeruginosa	negative	2	2	MDR
Klebsiella pneumoniae	negative	8	8	MDR, CARB-R
Staphylococcus aureus	positive	8	16	MRSA
Enterococcus faecalis	positive	8	16	VRE

Table S2. Minimum inhibitory concentration/minimum bactericidal concentration *E. coli* 25922, *P. aeruginosa* 27853, *K. pneumoniae* 13883, *S. aureus* 29213, and *E. faecalis* 29212. Minimum inhibitory concentration/minimum bactericidal concentration for resistant bacterial isolates of Gram-negative (*Acinetobacter baumannii*, *Pseudomonas aeruginosa*, and *Klebsiella pneumoniae*) and Gram-positive (*Staphylococcus aureus* and *Enterococcus faecalis*) bacteria.

Bacteria	Antibiotic	MIC (mg/L)	EUCAST Breakpoint (mg/L) S / R	EUCAST Interpretation
Acinetobacter baumannii	Colistin	64	S: ≤2 | R: >2	Resistant
Acinetobacter baumannii	Imipenem	64	S: ≤2 | R: >8	Resistant
Acinetobacter baumannii	Gentamicin	16	S: ≤4 | R: >4	Resistant
Acinetobacter baumannii	Amikacin	512	S: ≤16 | R: >16	Resistant
Acinetobacter baumannii	Meropenem	32	S: ≤2 | R: >8	Resistant
Acinetobacter baumannii	DNA-AgNCs (3HP)	6.5	—	—
Pseudomonas aeruginosa	Amikacin	512	S: ≤16 | R: >16	Resistant
Pseudomonas aeruginosa	Colistin	0.5	S: ≤2 | R: >2	Susceptible
Pseudomonas aeruginosa	Meropenem	256	S: ≤2 | R: >8	Resistant
Pseudomonas aeruginosa	DNA-AgNCs (3HP)	6.5	—	
Klebsiella pneumoniae	Colistin	>16	S: ≤2 | R: >2	Resistant
Klebsiella pneumoniae	Amikacin	>64	S: ≤8 | R: >16	Resistant
Klebsiella pneumoniae	Meropenem	>16	S: ≤2 | R: >8	Resistant
Klebsiella pneumoniae	Ceftazidime/Avibactam	8/2	S: ≤8 | R: >8	Susceptible
Klebsiella pneumoniae	Gentamicin	64	S: ≤2 | R: >4	Resistant
Klebsiella pneumoniae	DNA-AgNCs (3HP)	26	—	—
Enterococcus faecalis	Vancomycin	>32	S: ≤4 | R: >4	Resistant
Enterococcus faecalis	Linezolid	8	S: ≤4 | R: >4	Resistant
Enterococcus faecalis	Teicoplanin	64	S: ≤2 | R: >2	Resistant
Enterococcus faecalis	DNA-AgNCs(3HP)	26	—	—
Staphylococcus aureus	Eritromicin	>4	S: ≤1 | R: >2	Resistant
Staphylococcus aureus	Clindamicin	>2	S: ≤0.25 | R: >0.5	Resistant
Staphylococcus aureus	Daptomicin	>8	S: ≤1 | R: >1	Resistant
Staphylococcus aureus	Gentamicin	16	S: ≤1 | R: >1	Resistant
Staphylococcus aureus	Linezolide	>8	S: ≤4 | R: >4	Resistant
Staphylococcus aureus	Rifampicin	>4	S: ≤0.06 | R: >0.5	Resistant
Staphylococcus aureus	Oxacilin	>8	S: ≤2 | R: >2	Resistant
Staphylococcus aureus	DNA-AgNCs (3HP)	26	—	—

Table S3. Minimum inhibitory concentration (MIC) values multidrug-resistant Gram-negative bacterial isolates (*Acinetobacter baumannii, Pseudomonas aeruginosa,* and *Klebsiella pneumoniae*) and Gram-positive isolates (*Staphylococcus aureus* and *Enterococcus faecalis*) determined for standard antimicrobial agents and DNA-AgNCs (3HP). Interpretation according to EUCAST breakpoints 2026 (European Committee on Antimicrobial Susceptibility Testing, 2026; Version 16.0). S = Susceptible; R = Resistant.
